# Recurrent Tissue-Specific mtDNA Mutations Are Common in Humans

**DOI:** 10.1371/journal.pgen.1003929

**Published:** 2013-11-07

**Authors:** David C. Samuels, Chun Li, Bingshan Li, Zhuo Song, Eric Torstenson, Hayley Boyd Clay, Antonis Rokas, Tricia A. Thornton-Wells, Jason H. Moore, Tia M. Hughes, Robert D. Hoffman, Jonathan L. Haines, Deborah G. Murdock, Douglas P. Mortlock, Scott M. Williams

**Affiliations:** 1 Center for Human Genetics Research, Department of Molecular Physiology & Biophysics, Vanderbilt University School of Medicine, Nashville, Tennessee, United States of America; 2 Center for Human Genetics Research, Department of Biostatistics, Vanderbilt University School of Medicine, Nashville, Tennessee, United States of America; 3 Center for Human Genetics Research, Vanderbilt University School of Medicine, Nashville, Tennessee, United States of America; 4 Department of Biological Sciences, Vanderbilt University School of Medicine, Nashville, Tennessee, United States of America; 5 Dartmouth Medical School, Department of Genetics, Computational Genetics Lab, Lebanon, New Hampshire, United States of America; 6 Department of Pathology, Vanderbilt University School of Medicine, Nashville, Tennessee, United States of America; Stanford University School of Medicine, United States of America

## Abstract

Mitochondrial DNA (mtDNA) variation can affect phenotypic variation; therefore, knowing its distribution within and among individuals is of importance to understanding many human diseases. Intra-individual mtDNA variation (heteroplasmy) has been generally assumed to be random. We used massively parallel sequencing to assess heteroplasmy across ten tissues and demonstrate that in unrelated individuals there are tissue-specific, recurrent mutations. Certain tissues, notably kidney, liver and skeletal muscle, displayed the identical recurrent mutations that were undetectable in other tissues in the same individuals. Using RFLP analyses we validated one of the tissue-specific mutations in the two sequenced individuals and replicated the patterns in two additional individuals. These recurrent mutations all occur within or in very close proximity to sites that regulate mtDNA replication, strongly implying that these variations alter the replication dynamics of the mutated mtDNA genome. These recurrent variants are all independent of each other and do not occur in the mtDNA coding regions. The most parsimonious explanation of the data is that these frequently repeated mutations experience tissue-specific positive selection, probably through replication advantage.

## Author Summary

DNA mutations are expected to be formed randomly, thus any reproducible pattern of DNA somatic mutations across multiple individuals or even across organs within each individual is highly unexpected. Using next generation sequencing of multiple tissues from the same individuals we found several somatic mutations in mitochondrial DNA that appear in a heteroplasmic state in all individuals examined, but only in particular tissues. These mutations were only found in known regions of replication control for the mitochondrial DNA. These data imply the presence of tissue-specific positive selection for these variants.

## Introduction

Mitochondrial DNA (mtDNA) heteroplasmy is commonly thought to be the product of either maternal inheritance [1] or rare, random somatic mutations that undergo subsequent expansion within an individual via genetic drift [2], [3]. Inherited heteroplasmy should be present in many, but perhaps not all, tissues while somatic mutations spread only as a result of cell division subsequent to the mutation event. Somatic mutations will be restricted to those cells or tissues derived from a common progenitor, and should follow patterns of development. Such intra-individual patterns should therefore differ among individuals as a function of where and when the initial mutation occurred.

Under these standard models, both inherited and somatic heteroplasmies should differ among unrelated individuals. However, until recent advances in sequencing technology it was impossible to assay low levels of heteroplasmy across the entire mitochondrial genome. We applied massively parallel sequencing technology to test these models by deeply sequencing the same ten tissues from two unrelated individuals. Unexpectedly we found that certain tissues, notably kidney, liver and skeletal muscle, have recurrent mtDNA mutations that were undetectable in other tissues in the same individuals. These mutations were found across unrelated individuals in these same tissues. Neither the maternal inheritance nor the random somatic mutation models explain the observed patterns of recurrent mtDNA heteroplasmy. The common recurrence of these tissue-specific mutations indicates a completely different model of mtDNA heteroplasmy, namely a decidedly non-random process that results in particular mutations, but only in specific tissues.

## Results

### Sequencing, Coverage and Copy Number

Next generation sequencing provides several advantages over previous methods in that it allows detection of very low heteroplasmy levels across the entire mtDNA genome without having to target specific sites. We sequenced mtDNA from 10 tissues (kidney, lung, liver, small bowel, large bowel, skeletal muscle, spleen, brain white matter, skin above belt and skin below belt) obtained at autopsy from two cancer-free individuals (Text S1). In brief, mtDNA sequences were generated as 100 nt paired-end reads on Illumina HiSeq 2000 machines, were aligned to the human reference genome rCRS [4] using BWA [5], and then were locally realigned and recalibrated using GATK [6]. Variants were reported as heteroplasmic if their frequencies were ≥1% on both strands from reads with a mapping quality score ≥30. We further eliminated variants with any of the following artifacts: strand bias, low average base quality score, or clustering at read ends. Strand bias was evaluated at both base pair and motif levels. We identified 20 heteroplasmic sites with mutation levels >1% in our two subjects (Table 1).

**Table 1:** Complete list of heteroplasmic sites.

Position	60	64	66	67	72	94	189	203	408	414	564	10495	13274	13711	16034	16035	16036	16049	16093	16126
Reference	T	C	G	G	T	G	A	G	T	T	G	T	T	G	G	G	G	G	T	T
Alternative	C	A	A	A	C	A	G	A	A	G	A	C	C	A	A	A	A	A	C	C
Subject 1:																				
Skin-AB	-	-	-	-	-	-	-	-	-	1.7	-	-	-	-	-	-	-	-	94.3	-
Skin-BB	-	-	-	-	-	-	-	-	-	-	-	-	-	-	-	-	-	-	95.7	-
Brain-Gray	-	-	-	-	-	-	1.4	-	-	-	-	-	-	-	-	-	-	-	79.8	-
Brain-White	-	-	-	-	-	-	-	-	-	-	-	-	-	-	-	-	-	-	91.1	-
Spleen	-	-	-	-	-	-	-	-	-	-	-	-	-	-	-	-	-	-	90.4	-
Skel. Mus.	-	3.5	1.4	1.1	-	-	12.8	-	1.2	-	-	-	-	-	-	-	-	-	13.1	98.9
Heart	-	-	-	-	-	-	1.9	-	-	-	1.3	-	-	-	3.3	3.0	3.4	1.2	76.3	-
Sm. Bowel	-	-	-	-	-	-	-	-	-	-	-	-	-	-	-	-	-	-	94.9	-
Lg. Bowel	-	-	-	-	-	-	-	-	-	-	-	-	-	-	-	-	-	-	91.1	-
Liver	1.1	-	-	-	6.1	3.5	-	1.9	-	-	-	-	-	-	-	-	-	-	88.2	98.2
Kidney	1.3	-	-	-	5.3	3.6	-	-	-	-	-	-	-	-	-	-	-	-	93.9	-
Lung	-	-	-	-	-	-	-	-	-	-	-	-	-	-	-	-	-	-	88.9	-
Subject 2:																				
Skin-AB	-	-	-	-	-	-	-	-	-	-	-	-	-	-	-	-	-	-	-	-
Skin-BB	-	-	-	-	-	-	-	-	-	-	-	-	-	-	-	-	-	-	-	-
Brain-White	-	-	-	-	-	-	-	-	-	-	-	-	-	-	-	-	-	-	-	-
Spleen	-	-	-	-	-	-	-	-	-	-	-	-	1.2	-	-	-	-	-	-	-
Skel. Mus.	-	3.6	-	1.6	-	-	9.3	-	1.6	-	-	-	-	-	-	-	-	-	-	-
Sm. Bowel	-	-	-	-	-	-	-	-	-	-	-	-	-	-	-	-	-	-	-	-
Lg. Bowel	-	-	-	-	-	-	-	-	-	-	-	-	-	-	-	-	-	-	-	-
Liver	2.7	-	-	-	8.3	4.5	-	1.3	-	-	-	-	-	-	-	-	-	-	-	-
Kidney	3.5	-	-	-	11.0	4.0	-	-	-	-	-	-	-	-	-	-	-	-	-	-
Lung	-	-	-	-	-	-	-	-	-	-	-	-	-	-	-	-	-	-	-	-
BoneMarrow	-	-	-	-	-	-	1.5	-	-	-	-	1.3	-	1.3	-	-	-	-	-	-

The numbers are the percentages of non-reference alleles as defined by the Cambridge Reference Sequence, NC_012920.1. The “-” denotes no heteroplasmy detected above the threshold of 1%.

**Figure 1:**
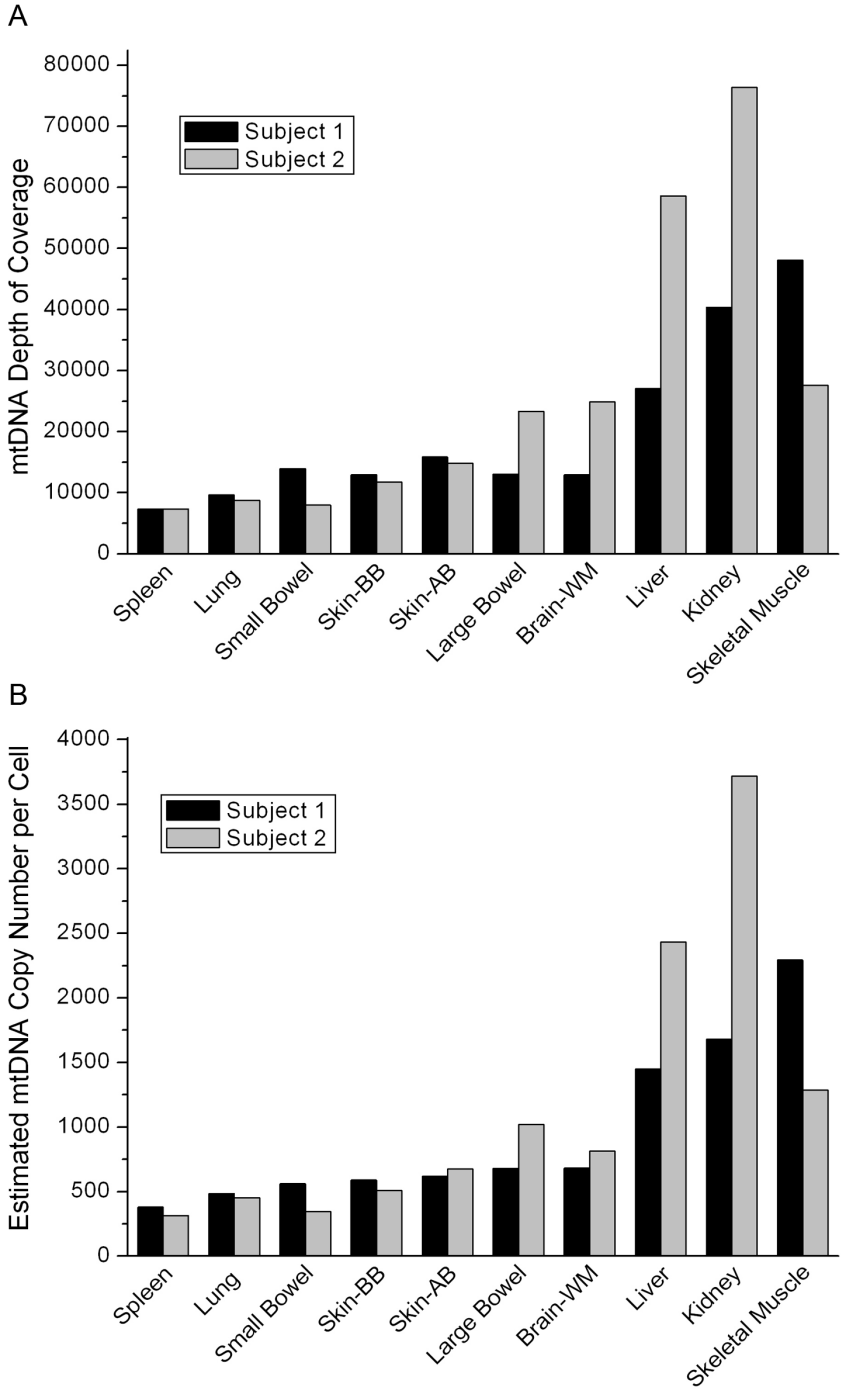
Coverage data. (A) The coverage of the mtDNA in each tissue in the two individuals sequenced in our study. (B) The mtDNA copy number per cell for each tissue estimated from the coverage data.

The coverage of mtDNA sequencing varied across tissues, ranging from over 7,000 to almost 80,000 (Figure 1A). By comparing mtDNA coverage to that for autosomal chromosomes, we estimated the mtDNA copy number per cell for each sample (Figure 1B). The two subjects had similar estimated mtDNA copy numbers in each tissue with values consistent with expectations based on previous data, ranging from a few hundred mtDNA per cell in spleen to a few thousand in the skeletal muscle [7], liver, and kidney.

Both subjects died of myocardial infarction and had no evidence of cancer or occult cancer. Subject 1 was a male, age 57 years, while subject 2 was a female, age 71 years (Table 2). This age and gender difference may explain the difference in mtDNA copy number in skeletal muscle between the two subjects.

**Table 2:** Demographics for our subjects.

	Subject 1	Subject 2	Subject 3	Subject 4
Gender	Male	Female	Male	Female
Age (years)	57	71	43	73
Race	White	White	White	White
Height (cm)	173	NA	178	NA
Weight (kg)	97.2	62.6	113	246
BMI	32.5	NA	35.6	NA
Tobacco usage	Yes	Negative	Negative	Negative
Drug usage	Negative	Negative	Negative	Negative
Alcohol consumption	Yes	Negative	Very little	Negative
Cause of Death	MI	MI	Accidental Death	Sepsis (pneumonia)

MI: myocardial infarction.

NA: Not available.

### Recurrence of Site-Specific Heteroplasmy

**Figure 2:**
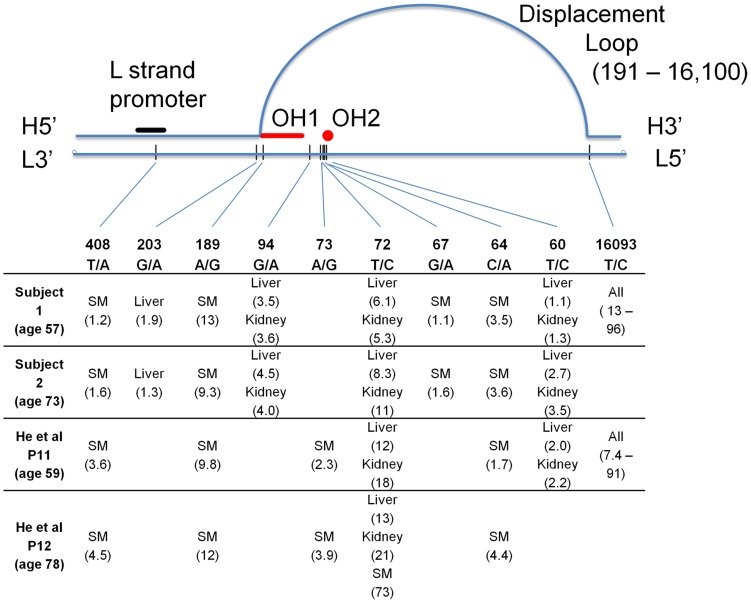
Graphical representation of the human mtDNA control region (sites 576-1 and 16569-16024). All sites found to be heteroplasmic in two or more subjects are listed, with measured percent heteroplasmy given after each tissue name. SM – skeletal muscle. H – heavy strand of mtDNA, L – light strand, OH1 and OH2 – origins of replication of the heavy strand. Site numbering is from rCRS.

Unexpectedly, we found eight sites to be heteroplasmic in the same tissues in both of our subjects (Figure 2). Contamination was unlikely since the pattern of these sites fit no known haplogroup, and the samples from the two subjects were collected at different times and sequenced at different facilities (see Materials and Methods). Most of these variants exist in the general population but are rare (Table S2) [8]. Some of these variants have been previously reported as being heteroplasmic in these same tissues but the recurrence and tissue-specificity of these mtDNA variations was not discussed [9]. In the four unrelated individuals combined from our study and that of He et al [9] there were 10 recurrent mutations. All of the recurrent mutations lay within the mtDNA control region, and nine of the ten recurrent mutations occurred in multiple individuals but only in specific tissues (site 16093 was the only exception and this mutation was found in a wide range of tissues). Six of the recurrent mutation sites were observed in both studies (Figure 2). Surprisingly, these recurrent tissue-specific mutations are all close to regulatory sites for mtDNA replication, indicating that these variations are likely to alter the replication dynamics of the mutated mtDNA molecules.

Three of the ten tissues studied (liver, kidney and skeletal muscle) harbored multiple mtDNA mutations that were shared across two or more of the four individuals combined from both studies (Figure 2). Mutations at sites 60 and 72 occurred in liver and kidney in both studies, while mutations at sites 94 and 203 were repeatedly detected in liver and/or kidney only in our subjects. Of these mutations, three sites (60, 72 and 94) when present in an individual, always occurred in both liver and kidney. Another three occurred in skeletal muscle of all four patients (sites 64, 189 and 408). Heteroplasmy at positions 189 and 408 has been found in skeletal muscle of the elderly [10]. A mutation at site 67 was found only in skeletal muscle in our two subjects. There was no observable linkage disequilibrium among the sites (all pairwise r^2^<0.007), indicating that these are independent mutations and are not due to contamination. Heteroplasmy for these tissue-specific recurrent mutations ranged from 1–21%, but was not detected in the other tissues. Strikingly, the level of heteroplasmy was similar across individuals in the same tissues (Figure 2). The depth of sequence coverage in these tissues (skeletal muscle, liver and kidney) ranged from 27,000–76,000× (Figure 1A), providing high confidence in our observation of heteroplasmy in these samples. Of the tissues we examined (with the notable exception of brain white matter), skeletal muscle, liver and kidney are the ones most often affected by mitochondrial disease [11].

Although the entire mtDNA genome was sequenced to high depth, we found no single base pair substitutions outside the control region that were repeated between individuals. The common 4977 deletion [12], [13], [14] was found in many tissues as expected. Here we have focused only on those heteroplasmic sites common to multiple subjects; the full list of identified heteroplasmic sites is given in Table 1. These data clearly indicate a non-random distribution (6.0e^−4^<p<6.0e^−6^) of recurrent heteroplasmic mutations that flank important regulatory elements for mtDNA replication (Figure 2). Several of the repeated variants were clustered (sites 60–72) near a recently reported origin of replication for the H strand [15], [16] at sites 54–57. Three of the other repeated mutations (189, 203 and 16093) occur very near the boundaries of the displacement loop [15]. Finally, site 408, which was heteroplasmic in the skeletal muscle of all four individuals, lies within the L strand promoter that initiates the RNA primer for mtDNA replication.

**Figure 3:**
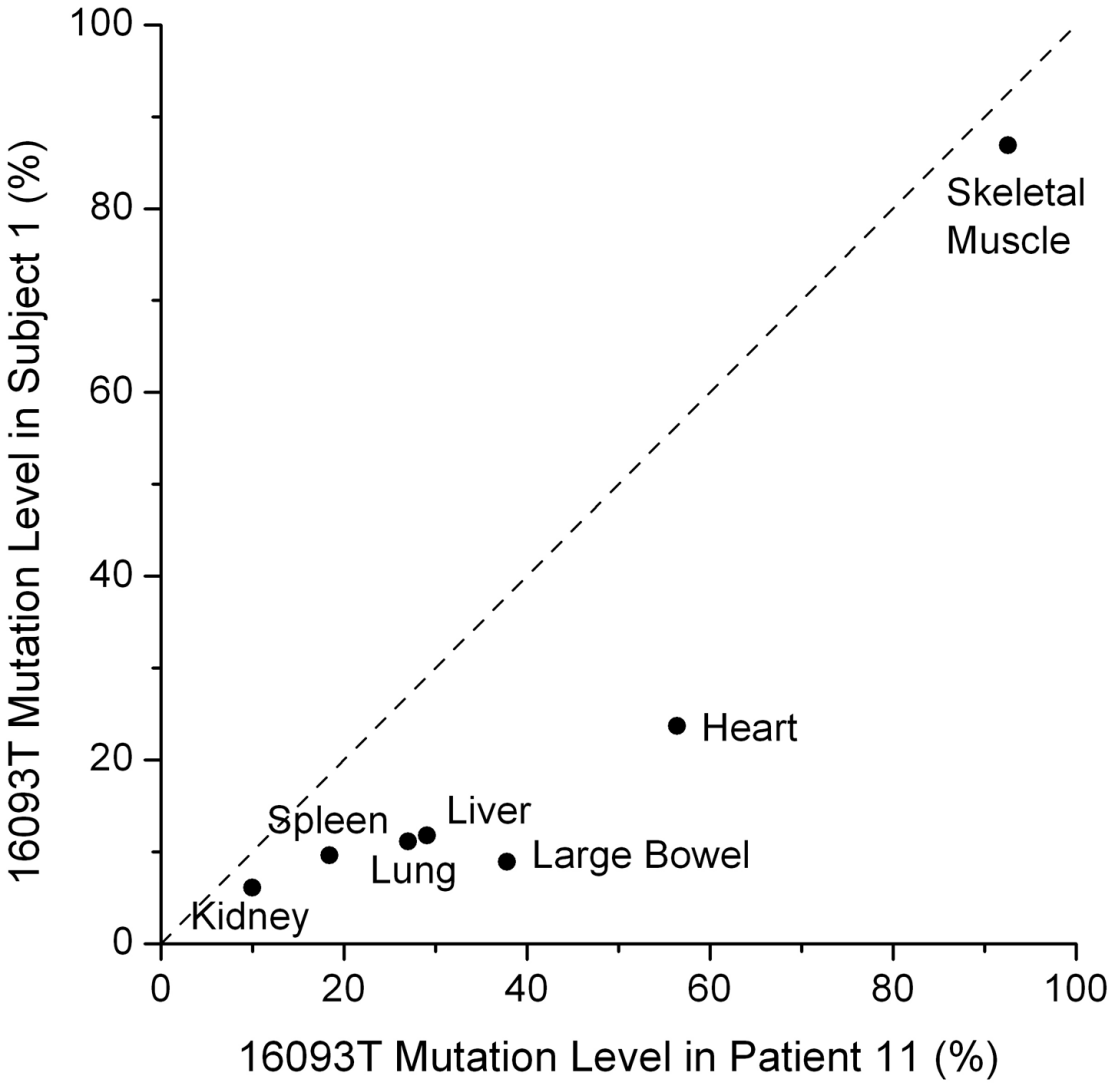
Tissue dependent heteroplasmy levels of the 16093 T/C variant across two subjects. The heteroplasmy levels in these two subjects have a correlation of 0.93 (p = 0.003). The diagonal line shows equal values in the two subjects. Patient 11 is from He *et al.* [9].

One variant, 16093, was unusual in that it was observed in all tested tissues in one subject from each study, with tissue-specific heteroplasmy levels that were strikingly similar across these two individuals (Figure 3; r = 0.93, p<0.003). These subjects were of similar age (59 and 57 years), so it is possible that the 16093 heteroplasmy increases with age at different rates in different tissues, leading to the similar heteroplasmy levels across tissues in these subjects. The 16093 site lies within a loop of a predicted large secondary structure of the mtDNA and is known to be hyper-mutable [17]. Individuals with the 16093C variant in blood tend to have C/T heteroplasmy in buccal cells [18]. In one of our subjects and in patient 11 from He *et al.* [9], the 16093C variant is the major allele in most tissues (except muscle), and these are the same two individuals who have widespread 16093T/C heteroplasmy across all tested tissues (Figure 2). The similar, tissue-dependent heteroplasmy levels at 16093 reinforce the observation that heteroplasmy levels at other sites are also comparable across individuals (Figures 2 and 3).

### Repeated Indel Mutations

We also found two insertion/deletion (indel) somatic mutations that were repeated across the two sequenced subjects in a tissue-specific pattern. Both are length variations in polynucleotide tracts. The human mtDNA reference sequence (rCRS) has a stretch of six guanines, denoted by G6, from sites 66–71. Both of our subjects had measurable heteroplasmy for the G5 variant, decreasing the length of this poly-G tract by one nucleotide. This variant was found in the same four tissues in both subjects: kidney (0.9% and 3.6% in subjects 1 and 2 respectively), large bowel (0.7% and 2.4%), small bowel (1.0% and 4.3%) and the white matter of the brain (1.9% and 3.6%). This poly-G tract is located adjacent to one of the recurrent heteroplasmic SNPs (Figure 2). In kidney, the G5 variant did not occur on the same reads as the heteroplasmic variant at site 72, demonstrating that this is not a sequencing artifact and that the variants are on different mtDNA molecules. Considering its location, it is reasonable to hypothesize that this poly-G length variant also affects mtDNA replication.

The second repeated heteroplasmic indel was in an 8-nucleotide poly-A tract at positions 12418–12425. This is the longest poly-A tract in the rCRS. In both subjects, we found the shorter A7 variant in the kidney samples only (1.0% heteroplasmy in subject 1 and 1.6% in subject 2). We also found the longer A9 variant in both kidney samples, but at very low levels (<1%). This indel is the only repeated mutation that we found in the coding region of the mitochondrial genome. It is located near the start of the MT-ND5 gene (12337–14148) and causes a frameshift mutation, severely altering almost the entire length of the ND5 protein, an essential component of complex I of the electron transfer chain.

### Molecular Validation and Replication

**Figure 4:**
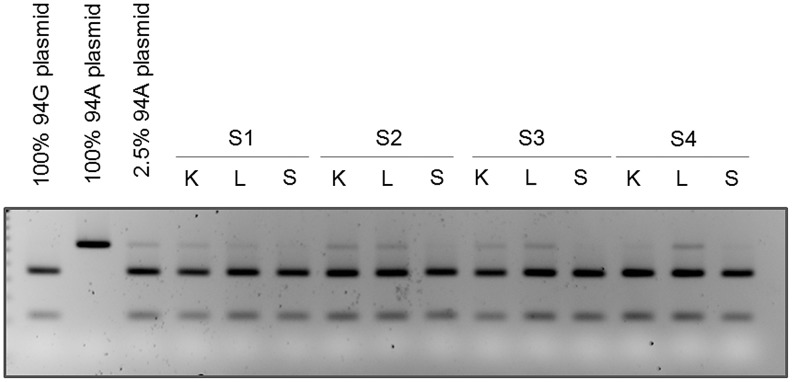
Mutation at position 94 is verified by RFLP analysis. DNA isolated from kidney (K), liver (L), and spleen (S) from subjects 1, 2, 3 and 4 were subjected to PCR and RFLP analysis. The presence of the mutation at position 94 is seen as the presence of the upper (uncut) band by gel electrophoresis. RFLP analysis of PCR products from wild type (94G), mutant (94A), and mixed (2.5% 94A) plasmid DNA are also loaded for comparison.

To confirm that the observed heteroplasmy was not due to sequencing artifact, we performed an alternative analysis for site G94A because it could be assayed using RFLP analysis. Specifically, the G allele at G94A permits digestion with the restriction enzyme BcoDI. Sensitivity of this assay was determined using a titration of plasmid constructs with and without the restriction site. This assay was specific to mtDNA because the primers did not amplify nuclear DNA (Figure S1A). We detected as low as 2.5% of the undigested variant (Figure S1B). RFLP analysis of all samples from the two sequenced individuals demonstrated that the tissues shown to be heteroplasmic from the sequencing analyses had both digested and undigested bands and were therefore heteroplasmic (Figure 4). As a negative control, we also analyzed this site in spleen DNA and found it to be consistent with the sequencing results (Figure 4). This result was statistically significant (p<0.001).

To test the generality of this heteroplasmy we examined two additional cancer-free individuals (Subjects 3 and 4 in Table 2) using the same RFLP assay. This analysis replicated heteroplasmy in liver in both additional subjects and in kidney in one individual. No significant heteroplasmy was detected in spleen in either subject, providing additional support for the tissue-specificity of this SNP (Figure 4). The result was also statistically significant (p<0.001).

## Discussion

### Previous Literature on the Recurrent Mutation Sites

Several of our observed heteroplasmic sites have been identified in studies of human disease. The T408A mutation, which was present in the muscle of all four sequenced individuals (two from the present study and two from He et al.), has been reported as an age-related somatic mutation in muscle [10], [19], [20], [21]. It has also been associated with disease in an investigation of a patient with a mitochondrial depletion syndrome [22] that was fatal at a young age (14 years), where the T408A mutation exhibited heteroplasmy at high levels (>70%) in all investigated maternal relatives, but was not detectible in the patient. The authors speculated that the T408A mutation interacted with a hypothesized nuclear DNA factor that affected mtDNA replication, thus leading to the mtDNA depletion in this patient. The A189G mutation, which was also present in the muscle of all four sequenced individuals, has also been reported in studies of aging muscle [10], [19], [20], [21], [23], [24], [25], and is often reported together with T408A. Both of these mutations increase in heteroplasmy level in muscle slowly with increasing age [19].

Heteroplasmy at site 16093 has often been reported in a range of tissues [18], [26], [27], [28], [29], [30], [31], [32], consistent with the observation of this variant being in all tissues in one of our two sequenced subjects and one of the subjects from He et al. The T414G mutation has been reported to accumulate with age in fibroblasts and skeletal muscle [33], [34] and we detected this mutation in one individual in a skin sample (Table 1). Our observation of heteroplasmy at 189, 408, 414, and 16093 and the previous studies reporting these same variants provide support for the validity of the next-generation sequencing data.

We found that four heteroplasmic somatic mutations (T60C, T72C, G94A, and G203A) recur, but only in liver and/or kidney. Given that liver and kidney arise from endoderm and mesoderm respectively, it is unlikely these mutations share a common developmental origin. These four sites are also global population polymorphisms, though at low frequencies [8] (Table S2). Some of these have been previously reported in the context of human disease. The T72C variant has been reported as a somatic mutation in the brain tissue of both Alzheimer's cases and controls [35], although it was not detected in any sequenced brain samples in this study. G94A has recently been reported in two Chinese pedigrees transmitting Lebers Hereditary Optic Neuropathy but in these families this variant was an inherited fixed polymorphism, not a heteroplasmic somatic mutation [36]. Despite the rarity of G203A in the global population (estimated as 0.3% in a survey of human mtDNA sequences deposited in GenBank) [8] it has been identified as a fixed variant in patients with deafness in two independent studies in different ethnicities [37], [38].

### Implications of the Recurrent Mutations

Our results indicate that mtDNA heteroplasmy due to somatic mutation is unexpectedly recurrent and tissue specific. By using a sensitive deep-sequencing technique across a wide range of tissues in multiple subjects we were able to test the hypothesis that specific mtDNA variations preferentially accumulate in particular tissues [34], [39]. One possible explanation for observing the same mtDNA heteroplasmic variants in two or more tissue types within the same person is that a mutation occurred early in embryonic development, before the tissues differentiated from their common progenitor. However, this hypothesis cannot explain the repeated observation of the same mutations in the same tissues in *unrelated* individuals. The occurrence of repeated mutations in the same tissues at sites that closely correspond to regulatory elements for mtDNA replication indicates somatic selection as the most likely mechanism driving the increase and maintenance of these heteroplasmic mutations. This inference is further supported by independent evidence that liver and kidney exhibit positive selection for mtDNA variants in a mouse model formed by artificially mixing the mtDNA of two different mouse strains [40], [41], [42]. In-vivo BrdU labeling in these mice over a time course of 50 hours did not detect any difference in labeling in liver samples between the two mtDNA haplotypes [41], leading the authors to conclude that replicative advantage was not the driving force for the segregation in these mice. However, we would argue that a 50 hour window is not comparable to the decades of replication advantage that would need to occur in our subjects. Recently, Sharpley et al [43] also generated a separate mouse model of heteroplasmy by mixing the naturally occurring NZB and 129S6 mtDNA sequences. This mouse model also showed that the segregation of the two mtDNA genomes varied in a tissue-specific manner, with liver and kidney having the strongest selection for the NZB version of the genome. Finally, liver has been argued to be under selection for nuclear aneuploidy and polyploidy, indicating that selection may have a special role in this tissue [44]. It is reasonable that the tissue specific selection of these mtDNA variants is due to regulation by nuclear-encoded mitochondrial genes with tissue-dependent expression, as has been shown in one of the mouse models in spleen [40]. In contrast to previous work documenting a wide variety of heteroplasmic sites and their functional implications, the unique value of this study is the comparison of mtDNA heteroplasmy across multiple tissues in several individuals and the demonstration that several somatic variants recur in a tissue-specific pattern.

The pattern of tissue-specific mutations we have found across multiple individuals could be explained by a few alternatives, including positive or negative selection. Under positive selection, mutations in certain tissues would increase in frequency due to their advantage. Under negative selection, mutations could occur at a high rate but would be removed from all tissues except for those where the recurrence is observed, where negative selection is presumably relaxed. Of these two alternatives, positive selection is the most likely explanation because under negative selection mutations should be scattered widely across the mtDNA control region, not just the recurrent ones at specific sites related to replication (Figure 2). In contrast, positive selection could simply be explained by a replication or other functional advantage in high copy number tissues due to increased mtDNA replication. It is important to note than any functional difference among the variant mtDNA molecules, if any, are due only to the sites we describe because all of our observed heteroplasmic sites are independent of each other, and there were no recurrent heteroplasmic sites in the coding regions.

Another alternative is that there are tissue-specific mutational hotspots within the mtDNA. For example, interferon-induced cytidine deaminases are capable of generating somatic mtDNA mutation in a tissue-specific fashion [45]. Although this alternative is not mutually exclusive to the selection argument, we still favor differential selection based on its simplicity, and on prior data suggesting that two of the tissues in which we observed recurrent mtDNA mutations, liver and kidney, also undergo selection in two separate heteroplasmic mouse models [40], [41], [42], [43]. The mouse models provide evidence suggesting that selection in the absence of any *de-novo* mutation generation can cause tissue specific heteroplasmy patterns because in both mouse models the two mtDNA haplotypes were artificially introduced through cell fusion and were not generated via a mutation process. Furthermore, even if the heteroplasmic sites we observed are mutational hotspots, their locations in the mtDNA genome are highly suggestive of functional roles (Figure 2). Homoplasmy in tumors has also been shown to possibly derive from random processes alone, but this computer simulation study is not directly analogous to recurrent heteroplasmy in normal tissues of multiple individuals [46]. Therefore, the most parsimonious and reasonable explanation for our data is positive selection in liver, kidney and skeletal muscle for certain mutations in and around the regions controlling mtDNA replication.

In a very different model system (i.e. a mouse strain with an abnormally high mutation rates due to a defective mtDNA polymerase) evidence for lower mutation load in the D loop was described [47]. Specifically, in this mouse model the accumulation of point mutations in the mtDNA was lower in the D loop region than the rest of the mtDNA. It is impossible to determine conclusively from these data whether this pattern is due to selection or to a variation in mutation rate, but it does demonstrate a non-random pattern in this part of the mtDNA, something we also observed but in a different direction.

Our data provide strong support for the conclusion that the current models of mtDNA variation are inadequate to explain what we now call “recurrent heteroplasmy”. The pattern of common, recurrent mutations we observed provides strong evidence that mtDNA heteroplasmy at several sites is non-random and is most likely the result of tissue-specific positive selection acting on the replication of mtDNA. The restriction of these mutations to liver, kidney and skeletal muscle indicates that the mtDNA replication process may vary across tissues, leading to tissue-specific selective forces, which correspond with high copy number tissues.

## Materials and Methods

### Tissue Collection

The protocols were approved by the Vanderbilt University Institutional Review Board. Samples were collected at autopsy within 48 hours post-mortem by a trained pathologist (RDH). Tissue samples for DNA extraction were collected using clean and sterile scalpels, placed in petri dishes, and transferred to 50 ml tubes containing ice-cold Dulbecco's phosphate-buffered saline (DPBS), rinsed again with DPBS and stored at −80°C until DNA extraction with exceptions as described below. Separate portions of the tissue sections were preserved in 10% formalin. Skin samples were collected from the ventral torso, from both above-belt (Skin-AB) and below-belt (Skin-BB) (e.g. above or below the waistline). Skeletal muscle was obtained from the diaphragm. The small and large bowel samples consisted of mucosal tissue that was collected by carefully scraping the loose mucosal layer from the internal surface of bowel sections. Bone marrow tissue was collected by flushing rib or vertebral body sections with DPBS and collecting the flushed material in a 50 ml tube on ice, which was then centrifuged to collect the cellular material. Splenocytes were isolated as previously described [48]. Gray and white brain samples were separated manually in a petri dish using a sterile scalpel. Demographic information for the four subjects is in Table 2.

### DNA Extraction

For each tissue two DNA extractions were performed. The tissue was lysed using the DNeasy Blood and Tissue Kit (Qiagen 69504). Once the tissue was lysed and incubated at 56°C overnight, one set of DNA extractions was transferred into a 2.0 ml tube (Sarstedt 72.694.406) and put on the QIAsymphony for automated extraction (Qiagen). The protocols used on the QIAsymphony were Tissue_LC_200_V5 and Tissue_HC_200_V5 depending on the tissue type. The second set of DNA extractions was transferred to Autopure Qubes D (Qiagen 949022) and 3 ml of Cell Lysis Solution (Qiagen 949006) was added to each tissue sample. These samples were then placed on the Autopure LS (Qiagen) for automated extraction. The protocol used on the Autopure was Cell Lysate. The resulting DNA was stored in Nunc Cryotubes (Nunc 377267). DNA from each protocol was calibrated and samples combined prior to sequencing.

### Sequencing and Data Processing

Sequences were generated as 100 nt paired-end reads on Illumina HiSeq 2000 machines. The two subjects were sequenced at different locations (subject 1 at Macrogen in South Korea and subject 2 at Illumina in California). Each sample was sequenced on 3–5 lanes, yielding 1.14–1.99 billion reads, which were aligned to the human reference genome hg19+rCRS (revised Cambridge Reference Sequence, NC_012920.1) using BWA [5] (ver. 0.5.9-r16). We performed local realignment and base quality score recalibration using GATK [6] (ver. 1.0.5974). The number of mapped reads ranged from 1.09–1.84 billion, with more than 90% of all reads being mapped except skeletal muscle from subject 2 (86.2%) (Table S1). Other programs were also used at various steps: samtools (ver. 0.1.16) for sorting and indexing bam files, bamtools (ver. 0.8.1025) for splitting and merging bam files, and picard (version 1.48) for marking duplicates and fixing mates after local realignment.

All samples were also genotyped on the Illumina Human Omni1 Quad chip with approximately one million SNPs in the nuclear genome. The consistency rate between the sequence- and chip-based SNP calls was >99.86% for all samples after standard quality control filtering. This indicates that the sequencing data were of high quality.

### Identification of Heteroplasmy and Testing for Artifacts Due to Non-Circularity of the Reference Genome

We screened for heteroplasmy in mtDNA using reads with MAPQ≥30. For each site, we calculated the fraction of bases A, C, G, T on the forward and reverse strands. A site was called heteroplasmic if it had ≥1% frequency for two or more bases on both strands and the variant did not have any of the following alignment artifacts: 1) strand bias, 2) clustering at read ends, and 3) low average base quality score. Due to the high read depth (Figure 1, Table S1) all duplicate reads were retained. Heteroplasmy estimates were assessed with and without the duplicate reads and heteroplasmy levels were not influenced by the duplicates.

The linear mtDNA reference genome (rCRS) was created by cutting the circular mtDNA at a fixed position. This may generate alignment artifacts near the linearization site (i.e., the ends of the mtDNA reference): 1) a read overlapping the linearization site may be unmapped or require heavy clipping to be aligned, 2) a read may be aligned but its paired-end mate may not be, and 3) a read may be aligned to one end of the reference but its mate to the other end. As a result, reads close to the linearization site may have low mapping quality scores and may be disproportionately filtered out. As our data are 100 nt reads with insert sizes mostly between 250 bp and 400 bp (Figure S2), these artifacts may influence the results hundreds of bases away from the mtDNA “ends”. To prevent artifacts due to mtDNA circularity we also created a new mtDNA reference genome by shifting the rCRS starting point to position 7002, and repeated the whole data processing steps as described above. Heteroplasmy was virtually identical between the two alignments, with less than 0.1% difference in heteroplasmy estimates.

In addition, the artificial N base at 3107 of the rCRS reference can lead to alignment artifacts. This N was removed before the alignment was made.

### Tests for Other Potential Sequencing Artifacts

For all observed heteroplasmic sites, we checked for various sequencing artifacts. The mtDNA control region harbors multiple poly-nucleotide tracts that could lead to sequencing artifacts. Since these artifacts often have strand bias, we filtered out all sites with strand bias. In addition, for each heteroplasmic site we performed a motif analysis similar to that described in detail for site 310 in the supplementary material, to identify artifacts (Table S4). We also checked for the presence of artifacts due to sequence similarities between the nuclear and mtDNA genomes (NUMT), and none could be detected (see Text S1). The reported sites are free of any artifacts.

### Distributions of Cycle and Strand for the Mutant Alleles

Alignment errors are known to cause artifacts that often show strand bias and excessive occurrence of mutant alleles at read ends. We found no cycle (i.e. position on the read) or strand bias for the mutant alleles at the heteroplasmic sites we identified. We extracted all bases with mapping quality score ≥30 and base quality score ≥20 at each heteroplasmic site and compared the distributions of cycle and strand for the major and mutant alleles. Figure S3 shows the distributions of C and A alleles for site 64 in the skeletal muscle of Subject 1. The mutant allele was uniformly distributed across the read length on both strands, showing no strand or cycle bias. Other heteroplasmic sites we identified had similar patterns.

### Levels of Linkage Disequilibrium between Heteroplasmic Sites

For heteroplasmic sites close enough to be on the same DNA read or read pair, we assessed whether the minor alleles are on the same haplotype background, or in other words, if they are in linkage disequilibrium (LD). LD between the variants could be a sign of either contamination or sequencing artifacts. Specifically, for every pair of sites ≤100 bp apart (e.g., 60-72-94 in liver and kidney tissues and 64–67 in skeletal muscles), we extracted reads that covered both positions and had MAPQ≥30. We further required that the reads had all bases matched (i.e., CIGAR string “100M”) or had clipping at one end (i.e., CIGAR string matching the regular expression pattern “[0–9]*S[0–9]*M” or “[0–9]*M[0–9]*S”), and the bases at the two sites had base quality score ≥20. We then tallied haplotypes and calculated r^2^ between the two sites. All r^2^ values were very close to zero (<0.004), indicating no LD between any heteroplasmic sites.

For every pair of sites >100 bp apart (e.g. between 60-72-94 and 203 in liver tissues; among 64–67, 189, and 408 in skeletal muscles), we extracted read pairs that covered both positions and had MAPQ≥30 and then followed the above procedure. Again, all r^2^ values were very close to zero (<0.007), indicating no LD in the mtDNA.

### Sequencing Error Rate

The sequencing error rate is reflected in the recalibrated base quality scores. For example, a base quality (BQ) score of 25 means the error rate for that base is 0.32%, BQ = 27 means 0.2%, and BQ = 30 means 0.1%. These error rates are much lower than the 1% detection cutoff we used for the determination of heteroplasmic sites. Figure S4 shows the distribution of recalibrated base quality scores. For all our samples, 82.4% bases had recalibrated base quality score ≥30, 91.4% had scores ≥27, and 94.4% had scores ≥25. These results provide assurance that the bases we used for our inferences had high quality and an error rate much lower than our heteroplasmy detection threshold.

### Estimation of mtDNA Copy Number

We calculated the depth of coverage for autosomes and mtDNA as:
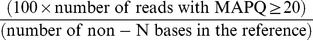
The multiplier 100 was used because we had 100 nt reads. The mtDNA depth ranged from 5651×–119203×, and the autosome depth ranged 37×–61× (Table S1). Assuming each cell carries a diploid (2×) nuclear genome, the mtDNA copy number was estimated as:
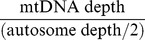
The estimated mtDNA copy number ranged from 315 to 5880 (Table S1).

### Statistical Tests

To test for non-randomness of the recurrent mutations, we calculated the probability that a mutation occurred at these 10 sites under two extreme scenarios. Using a model of constant mutation rate, *c*, along the whole mtDNA genome, the probability for a mutation to occur anywhere would be 16569*c* and the probability for it to occur at these 10 sites would be 10*c*. Thus the probability for an observed mutation in a DNA sample to occur only at any of these 10 sites is 10/16569 = 6.0e^−4^. Now suppose a mutation has been observed in a specific tissue of an individual. The probability to observe the same mutation in another individual only in the same tissue (out of 10 tissues) and on the same site (out of 10 sites) is further reduced to 6.0e^−6^. The recurrence patterns of the reported mutations fall between these two extreme scenarios, and therefore their p-values are between 6.0e^−4^ and 6.0e^−6^.

To test for correlation of 16093 heteroplasmy levels between the two individuals (Figure 3), we calculated the Pearson correlation coefficient and its associated p-value. The correlation was 0.93 and the p-value was 0.0028.

We calculated the p-value to evaluate the significance of our RFLP validation and replication results. For the validation part, we performed RFLP on six tissues (kidney, liver, spleen from Subjects 1–2). Let *a* = P(detect 94A|94A is absent), the probability of falsely detecting 94A in RFLP analysis while it was absent. Then the probability of seeing 94A in two kidneys, two livers but not the two spleens is *a* ^4^(1−*a*)^2^. The value of *a* is probably lower than the false positive rate for sequencing analysis, which would be at most 0.2 (4 out of 20 tissues when the sequencing results were assumed to be false). Even at *a* = 0.2, the p-value will be *a* ^4^(1−*a*)^2^ = 0.001. The p-value will be much smaller at a lower value of *a*; for example, *p* = 5.6e^−6^ if *a* = 0.05, and *p* = 9.8e^−9^ if *a* = 0.01. The p-value for the replication part can be similarly evaluated.

### Haplogroup Determination

We determined the mtDNA haplogroups for our subjects: T2a1 for subject 1 and H1a1 for subject 2 (Table S3). Haplogrouping was performed using the H-Mito program (http://www.phylotree.org) supplied by Mannis van Oven [49].

### RFLP Analysis

To provide molecular validation of sequencing results, we performed RFLP analysis using control plasmids and patient DNA from suspected heteroplasmic and homoplasmic tissues. We focused on position 94, which sequencing results identified as heteroplasmic in kidney and liver. Control samples consisted of 100% wild-type plasmids at position 94 (G), 100% mutant plasmids (A), or 97.5% wild-type and 2.5% mutant plasmids. DNA samples from suspected heteroplasmic kidney and liver tissues, and from suspected homoplasmic spleen tissue were analyzed for subjects 1–4. Fifteen nanograms of the control plasmid or patient DNA was amplified with 10 µM D-loop-targeted forward (5′-GATCACAGGTCTATCACCCTATTAAC-3′) and reverse (5′-CAGATACTGCGACATAGGGTGCT-3′) primers (Operon) and Platinum PCR Supermix (Invitrogen) according to manufacturer's directions. Following amplification, PCR products were digested for 8 h at 37°C with the restriction enzyme BcoDI, which cuts the wild-type but not mutant PCR product at position 94. Successful digestion resulted in cutting of the 130-bp PCR product into 90- and 40-bp fragments. We added 5 µL 5× gel-loading dye (KD Medical/MediaTech) to each 20-uL reaction after restriction digest, and loaded 12 µL of digest products and gel-loading dye into each well of a 3% agarose (Sigma) gel in 1× TBE (Cellgro) with 0.0125% ethidium bromide (Bio-Rad). The gel was run at 150 V for 2 h, then UV imaged for 200 ms in a Syngene G:Box imager.

## Supporting Information

Figure S1 RFLP analysis can detect low levels of mutation at position 94. (A) PCR using primers surrounding position 94 of the D loop of the mtDNA amplify a 130 bp fragment from HeLa cell or human brain total DNA. No amplification is seen when rho zero (lacking mtDNA) cell DNA is used as template, indicating no amplification from nuclear mtDNA insertions (nuMTs). (B) Sensitivity of RFLP analysis. Mixtures of plasmids containing a wild type (94G) or mutant (94A) allele were used to determine the sensitivity of the RFLP analysis. Plasmid mixtures were subjected to PCR and the amplified DNA fragments were digested with BcoDI.
(DOCX)

Figure S2 Distribution of mtDNA insert size for subject 1 (left) and subject (right). To address these issues, we created a new mtDNA reference genome, starting at position 7002 and without the N base at 3107. We then aligned all reads, using hg19 and this new mtDNA reference to compare with the alignment to the original rCRS. The list of heteroplasmic sites was the same for both alignments. Heteroplasmy levels were estimated using the alignment for which the linearization site was more distant from the evaluated site. All heteroplasmic sites had only two alleles with ≥1% frequency and one of the two alleles was always the reference allele in the rCRS.
(DOCX)

Figure S3 Distributions of cycle for bases C and A at site 64 in the skeletal muscle of Subject 1.
(DOCX)

Figure S4 Distribution of base quality score after recalibration for subject 1 (left) and subject 2 (right).
(DOCX)

Table S1 Summary of sequencing
(DOCX)

Table S2 Sequence properties of the recurrent heteroplasmic sites
(DOCX)

Table S3 Haplogroup information for our subjects: Alleles at non-heteroplasmic sites that differ from the rCRS reference are shown. The alleles are fixed in all tissues except for 16126, which was heteroplasmic in two tissues of Subject 1 (Table 1). Site 16093 is also included because its major allele is not the reference allele in all but one tissue of Subject 1.
(DOCX)

Table S4 Variations in the region around site 310 in the skeletal muscle sample from Subject 1. Motifs with less than 10 copies are not shown. Bases that differ from the major motif are in red.
(DOCX)

Text S1 Supplemental data and description of methods.
(DOC)
