# Supplementary figures and images for: Recurrent Tissue-Specific mtDNA Mutations Are Common in Humans
Source: PLoS Genet. 2013 Nov 7;9(11):e1003929. doi: 10.1371/journal.pgen.1003929 (PMC3820769; doi:10.1371/journal.pgen.1003929)

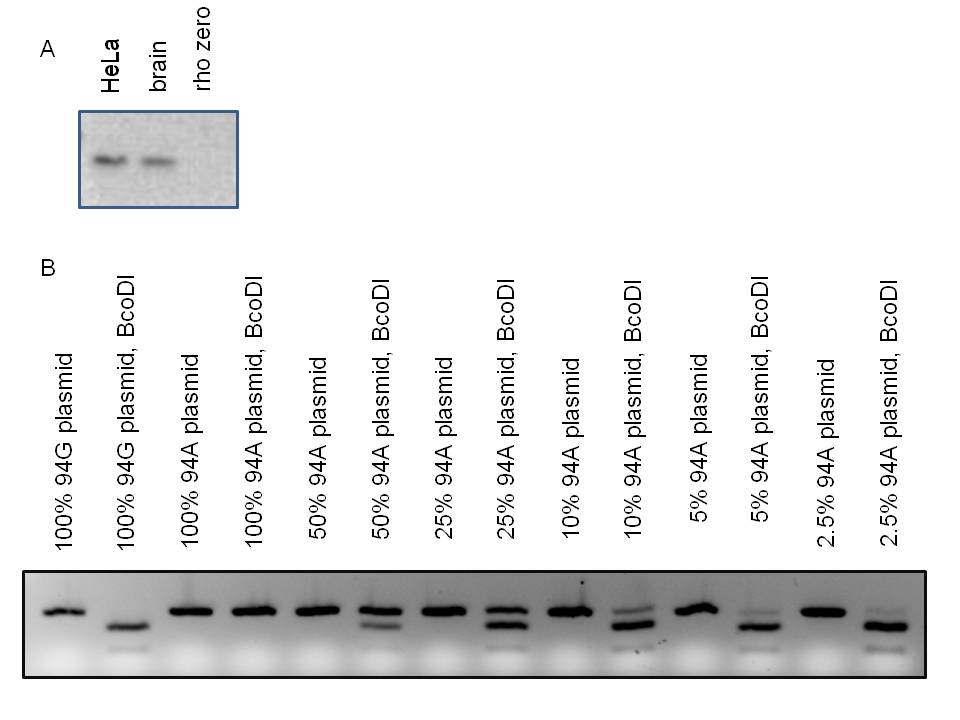

Supplement: Figure S1 — RFLP analysis can detect low levels of mutation at position 94. (A) PCR using primers surrounding position 94 of the D loop of the mtDNA amplify a 130 bp fragment from HeLa cell or human brain total DNA. No amplification is seen when rho zero (lacking mtDNA) cell DNA is used as template, indicating no amplification from nuclear mtDNA insertions (nuMTs). (B) Sensitivity of RFLP analysis. Mixtures of plasmids containing a wild type (94G) or mutant (94A) allele were used to determine the sensitivity of the RFLP analysis. Plasmid mixtures were subjected to PCR and the amplified DNA fragments were digested with BcoDI. (DOCX) [file pgen.1003929.s001.docx]

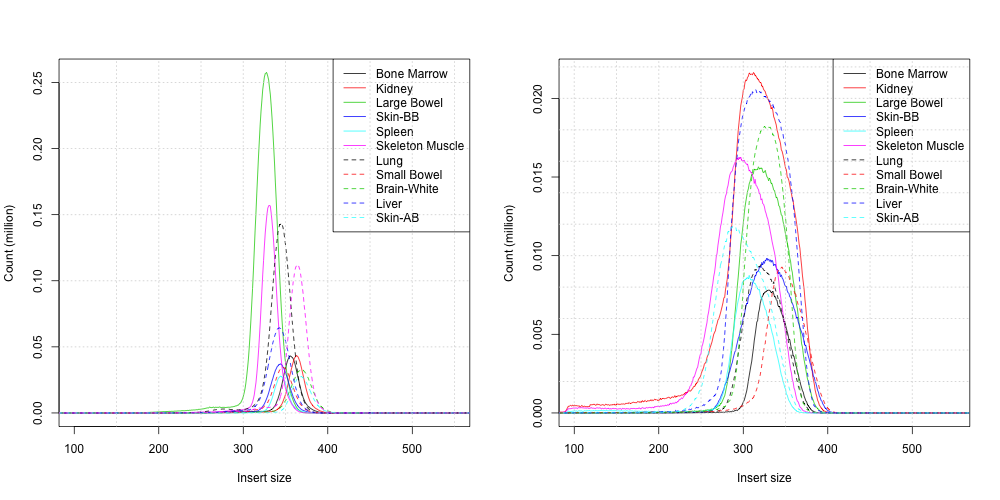

Supplement: Figure S2 — Distribution of mtDNA insert size for subject 1 (left) and subject (right). To address these issues, we created a new mtDNA reference genome, starting at position 7002 and without the N base at 3107. We then aligned all reads, using hg19 and this new mtDNA reference to compare with the alignment to the original rCRS. The list of heteroplasmic sites was the same for both alignments. Heteroplasmy levels were estimated using the alignment for which the linearization site was more distant from the evaluated site. All heteroplasmic sites had only two alleles with ≥1% frequency and one of the two alleles was always the reference allele in the rCRS. (DOCX) [file pgen.1003929.s002.docx]

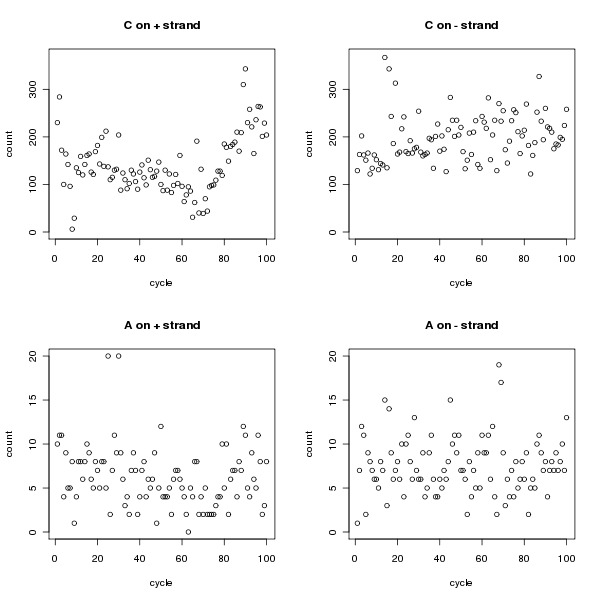

Supplement: Figure S3 — Distributions of cycle for bases C and A at site 64 in the skeletal muscle of Subject 1. (DOCX) [file pgen.1003929.s003.docx]

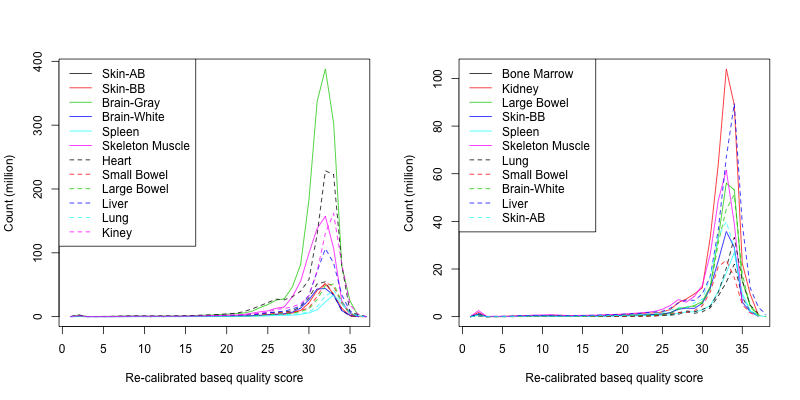

Supplement: Figure S4 — Distribution of base quality score after recalibration for subject 1 (left) and subject 2 (right). (DOCX) [file pgen.1003929.s004.docx]
