# Supplementary material for: Recurrent Tissue-Specific mtDNA Mutations Are Common in Humans
Source: PLoS Genet. 2013 Nov 7;9(11):e1003929. doi: 10.1371/journal.pgen.1003929 (PMC3820769; doi:10.1371/journal.pgen.1003929)
Supplement: Table S1 — Summary of sequencing (DOCX) [file pgen.1003929.s005.docx]

| **Sample ID** | **Tissue** | **#reads (billion)** | **#mapped (billion)** | **Mapped rate** | **Autosome depth (MQ≥20)** | **mtDNA depth (MQ≥20)** | **mtDNA copy number** |
| --- | --- | --- | --- | --- | --- | --- | --- |
| **Subject 1:** |  |  |  |  |  |  |  |
| 200102630 | Skin-AB | 1.59 | 1.50 | 0.943 | 51.2 | 15859 | 619 |
| 200102632 | Skin-BB | 1.34 | 1.28 | 0.956 | 44.0 | 12946 | 589 |
| 200102634 | Brain-Gray | 1.26 | 1.20 | 0.956 | 40.5 | 119203 | 5880 |
| 200102636 | Brain-White | 1.16 | 1.11 | 0.956 | 37.9 | 12928 | 681 |
| 200102638 | Spleen | 1.17 | 1.12 | 0.961 | 38.7 | 7353 | 379 |
| 200102640 | Skel. Muscle | 1.29 | 1.23 | 0.954 | 42.0 | 48106 | 2292 |
| 200102642 | Heart | 1.63 | 1.54 | 0.944 | 52.4 | 64725 | 2468 |
| 200102644 | Small Bowel | 1.53 | 1.45 | 0.950 | 49.6 | 13970 | 562 |
| 200102646 | Large Bowel | 1.17 | 1.12 | 0.956 | 38.4 | 13046 | 679 |
| 200102648 | Liver | 1.14 | 1.09 | 0.955 | 37.4 | 27089 | 1450 |
| 200102652 | Kidney | 1.47 | 1.41 | 0.956 | 48.0 | 40378 | 1680 |
| 200102650 | Lung | 1.20 | 1.15 | 0.961 | 39.7 | 9630 | 485 |
| **Subject 2:** |  |  |  |  |  |  |  |
| SS6002950 | Skin-AB | 1.38 | 1.31 | 0.946 | 43.9 | 14870 | 677 |
| SS6002877 | Skin-BB | 1.53 | 1.39 | 0.909 | 46.2 | 11746 | 508 |
| SS6002945 | Brain-White | 1.99 | 1.84 | 0.925 | 61.2 | 24901 | 813 |
| SS6002941 | Spleen | 1.52 | 1.40 | 0.921 | 46.6 | 7347 | 315 |
| SS6002942 | Skel. Muscle | 1.52 | 1.31 | 0.862 | 42.8 | 27557 | 1286 |
| SS6002944 | Small Bowel | 1.48 | 1.38 | 0.928 | 45.9 | 7973 | 347 |
| SS6002875 | Large Bowel | 1.50 | 1.38 | 0.919 | 45.7 | 23350 | 1020 |
| SS6002949 | Liver | 1.61 | 1.46 | 0.906 | 48.2 | 58626 | 2434 |
| SS6002871 | Kidney | 1.36 | 1.25 | 0.913 | 41.1 | 76343 | 3718 |
| SS6002943 | Lung | 1.22 | 1.15 | 0.941 | 38.7 | 8767 | 453 |
| SS6002867 | Bone Marrow | 1.35 | 1.24 | 0.921 | 41.5 | 5651 | 272 |
|  |  |  |  |  |  |  |  |
