# Supplementary material for: Recurrent Tissue-Specific mtDNA Mutations Are Common in Humans
Source: PLoS Genet. 2013 Nov 7;9(11):e1003929. doi: 10.1371/journal.pgen.1003929 (PMC3820769; doi:10.1371/journal.pgen.1003929)
Supplement: Table S2 — Sequence properties of the recurrent heteroplasmic sites (DOCX) [file pgen.1003929.s006.docx]

| Site | Sequence context | Notes | GenBank frequency (homoplasmic variations) |
| --- | --- | --- | --- |
| 60 | TTTGGTATTT**T**CGTCTGGGGG | End of short T tract | 0.02% |
| 64 | GTATTTTCGT**C**TGGGGGGTAT | Near long G tract | 2.5% |
| 67 | TTTTCGTCTG**G**GGGGTATGCA | In long G tract | Not observed |
| 72 | GTCTGGGGGG**T**ATGCACGCGA | Near long G tract | 1.8% |
| 94 | AGCATTGCGA**G**ACGCTGGAGC | No obvious features | 0.4% |
| 189 | TTACAGGCGA**A**CATACTTACT | No obvious features | 4.8% |
| 203 | ACTTACTAAA**G**TGTGTTAATT | 3xGT repeat | 0.3% |
| 408 | TTTTATCTTT**T**GGCGGTATGC | Short T tract | 0.2% |
| 16093 | CGCTATGTAT**T**TCGTACATTA | Known highly mutated position | 4.9% |
