# Supplementary material for: Recurrent Tissue-Specific mtDNA Mutations Are Common in Humans
Source: PLoS Genet. 2013 Nov 7;9(11):e1003929. doi: 10.1371/journal.pgen.1003929 (PMC3820769; doi:10.1371/journal.pgen.1003929)
Supplement: Table S3 — Haplogroup information for our subjects: Alleles at non-heteroplasmic sites that differ from the rCRS reference are shown. The alleles are fixed in all tissues except for 16126, which was heteroplasmic in two tissues of Subject 1 (Table 1). Site 16093 is also included because its major allele is not the reference allele in all but one tissue of Subject 1. (DOCX) [file pgen.1003929.s007.docx]

| rCRS position | Reference | Subject 1 | Subject 2 |
| --- | --- | --- | --- |
| 73 | A | G | G |
| 263 | A | G | G |
| 709 | G | A |  |
| 750 | A | G | G |
| 1438 | A | G | G |
| 1888 | G | A |  |
| 2706 | A | G |  |
| 2850 | T | C |  |
| 3010 | G |  | A |
| 4216 | T | C |  |
| 4769 | A | G | G |
| 4917 | A | G |  |
| 6365 | T |  | C |
| 7022 | T | C |  |
| 7028 | C | T |  |
| 8860 | A |  | G |
| 8697 | G | A |  |
| 8860 | A | G |  |
| 10463 | T | C |  |
| 11251 | A | G |  |
| 11719 | G | A |  |
| 11812 | A | G |  |
| 13368 | G | A |  |
| 13965 | T | C |  |
| 14233 | A | G |  |
| 14687 | A | G |  |
| 14766 | C | T |  |
| 14905 | G | A |  |
| 15326 | A | G | G |
| 15452 | C | A |  |
| 15607 | A | G |  |
| 15928 | G | A |  |
| 16093 | T | C |  |
| 16126 | T | C |  |
| 16162 | A |  | G |
| 16209 | T |  | C |
| 16294 | C | T |  |
| 16296 | C | T |  |
| 16519 | T | C | C |
| Haplogroup |  | T2a1 | H1a1 |
