# Supplementary material for: Recurrent Tissue-Specific mtDNA Mutations Are Common in Humans
Source: PLoS Genet. 2013 Nov 7;9(11):e1003929. doi: 10.1371/journal.pgen.1003929 (PMC3820769; doi:10.1371/journal.pgen.1003929)
Supplement: Table S4 — Variations in the region around site 310 in the skeletal muscle sample from Subject 1. Motifs with less than 10 copies are not shown. Bases that differ from the major motif are in red. (DOCX) [file pgen.1003929.s008.docx]

| Motif | Positive strand count | Negative strand count |
| --- | --- | --- |
| Legitimate motifs: |  |  |
| AACCCCCCCTCCCCCCGCTTC | 6588 | 7328 |
| AA**C**CCCCCCCTCCCCCCGCTTC | 79 | 75 |
| Sequencing artifacts: |  |  |
| AACCCCCCC**C**CCCCCCGCTTC | 38 | 279 |
| AACCCCCCCTCCCCCC**C**CTTC | 226 | - |
| AACCCCCCCTCCCCCCGC**C**TC | 123 | - |
| AACCCCCCCTCCCCCC**C**C**C**TC | 34 | - |
| AACCCCCCCTCCCCCCGCT**C**C | 27 | - |
| AACCCCCCCTCCCCCC**T**CTTC | 13 | - |
| AACCCCCCCTCCCCCCGC**G**TC | 12 | - |
| AACCCCCCCTCCCCC**A**GCTTC | 11 | - |
| A**C**CCCCCCCTCCCCCCGCTTC | - | 2243 |
| A**C**CCCCCCC**C**CCCCCCGCTTC | - | 559 |
| A**G**CCCCCCCTCCCCCCGCTTC | - | 95 |
| A**T**CCCCCCCTCCCCCCGCTTC | - | 91 |
| **CC**CCCCCCC**C**CCCCCCGCTTC | - | 61 |
| A**CC**CCCCCCCTCCCCCCGCTTC | - | 39 |
| AACCCCCCC**G**CCCCCCGCTTC | - | 35 |
| AACCCCCCC**A**CCCCCCGCTTC | - | 32 |
| **CC**CCCCCCCTCCCCCCGCTTC | - | 28 |
| **C**CCCCCGCTTC | - | 23 |
| A**C**CCCCCCC**G**CCCCCCGCTTC | - | 20 |
| A**G**CCCCCCC**C**CCCCCCGCTTC | - | 16 |
| A**T**CCCCCCC**C**CCCCCCGCTTC | - | 16 |
| **C**ACCCCCCCTCCCCCCGCTTC | - | 13 |
|  |  |  |
