# Supplementary material for: Recurrent Tissue-Specific mtDNA Mutations Are Common in Humans
Source: PLoS Genet. 2013 Nov 7;9(11):e1003929. doi: 10.1371/journal.pgen.1003929 (PMC3820769; doi:10.1371/journal.pgen.1003929)
Supplement: Text S1 — Supplemental data and description of methods. (DOC) [file pgen.1003929.s009.doc]

**SUPPLEMENTAL INFORMATION**

**Sequence context and population prevalence of the recurrent tissue-specific mutations.**

Table S2 gives the flanking sequence (from the rCRS) for each of the recurrent tissue specific mutations detected in this study. The frequencies for these sites as homoplasmic variants in the global population were based on a survey of human mtDNA sequences from GenBank .

**Power of detecting heteroplasmy**

The power to detect heteroplasmy is very high with the read depth in our data. For example, using a binomial model at depth 10000, with our 1% detection cutoff, we have 95% power to detect a heteroplasmy level of 1.18%, 99% power for 1.26% heteroplasmy, and 99.9% for 1.34% heteroplasmy. At depth 5000, we have 95% power to detect a heteroplasmy level of 1.27%, 99% power for 1.38% heteroplasmy, and 99.9% for 1.52% heteroplasmy.

**Heteroplasmy and sequencing artifacts around site 310**

It has been difficult to study the region surrounding site 310 because it is flanked by two C tracts (e.g., this region was excluded from analysis in He *et al*. ). The 310 reference allele is T in the rCRS, with the surrounding sequence being AACCCCCCCTCCCCCGCTTC from 301 to 320 (abbreviated as C_7_TC_5_). Both of our subjects had C_7_TC_6_ at about 1% frequency, showing an insertion of C to the right of 310 as the major motif in all tissues. The long C tracts can lead to sequencing artifacts that might appear as heteroplasmies. To address this issue, we analyzed this region in detail.

For each sample, we took all reads that had MAPQ ≥ 30 and matched the flanking sequence CC[A/C]CCA to the left and TGGCCACAG to the right, and extracted the sequence (called a motif below) between these two flanking sequences. Table S4 shows the results for skeletal muscle of subject 1. These extracted sequences are not at read ends because the read must have extra bases to the left and right to match the above flanking sequences. It is clear that the major motif was C_7_TC_6_. Among the alternative motifs, C_8_TC_6_ was present on both strands with approximately 1% heteroplasmy. The mutation T310C was also on both strands but had a strong strand bias. The other alternative motifs exhibited strong sequencing artifacts that are characteristic of mononucleotide repeats: those on the forward strand differed from the major motif at the end of the second C tract, and those on the reverse strand differed from the major motif at the end of both C tracts. Sites with large strand bias were considered to be artifacts (Table S4). All other samples had the same pattern (results not shown).

**Three other tissue samples in our data**

In addition to the ten tissues presented in the paper, we collected DNA samples from brain gray matter (brain-gray) and heart tissues of subject 1 and from bone marrow of subject 2. Results from these three tissues were not discussed in the main text because we did not have matching tissue samples from our two subjects nor in the two sequenced subjects in He et al. All three tissues have heteroplasmy 1.4-1.9% at position 189 (Table 1), the site at which skeletal muscles from all four subjects showed heteroplasmy (Figure 2).

For subject 1, both brain-gray and heart tissues had heteroplasmy at 16093 (Table 1), which is consistent with all other tissues. In addition, the heart tissue had 1.2-3.4% heteroplasmy at 564, 16034, 16035, 16036, and 16049; and the bone marrow tissue had 1.3% heteroplasmy at 10495 and 13711. The heart tissue also had a high mtDNA copy number (Table S1).

The brain-gray tissue had an estimated mtDNA copy number of 5880 while brain-white had only 681 in subject 1 (Table S1). The brain-white samples in both subjects had comparable mtDNA copy numbers, 681 and 813, respectively for subjects 1 and 2.

**Heteroplasmic sites not observed in He et al.**

Three sites (67, 94, 203) had heteroplasmy in our subjects but were not observed in the two subjects in He *et al.*  (Figure 2). This could be due to multiple factors. There is a potential variation of mutation patterns among the subjects. In He *et al.*, mtDNA was captured from total cellular DNA prior to library construction, which might result in increased variation in coverage compared to our data and a subsequent lack of sufficient depth for certain sites. The sequencing data in He *et al.* were only 36nt single-end reads, while our data were generated as 100nt paired-end reads. The much longer reads in pairs improve the quality of alignment.

One subject in He *et al.* had very high heteroplasmy level (73%) at site 72 in skeletal muscle. This was not observed in skeletal muscle of the other subject in their data, nor was it observed in the skeletal muscles of our two subjects (Figure 2). In addition, site 73 had heteroplasmy in skeletal muscles of both subjects in He *et al*. but not in ours. As shown in Table S2, both sites are at the end of a 6-base G tract that could lead to sequencing or alignment errors. These errors can be filtered out with long paired-end reads and stringent filtering criteria.

**NUMT regions**

NUMT regions, part of the nuclear genome, share high sequence similarity with some mtDNA regions.  Therefore, it is possible that mtDNA reads have been mapped to the NUMT regions and vice versa.  We have carefully evaluated the potential artifact due to this and concluded there is no effect on our results. Since each cell carries hundreds or thousands of copies of the mitochondrial genome and only two copies of the nuclear genome, the effect of NUMT reads misaligned to mtDNA is expected to be negligible in our data set. On the other hand, if mtDNA reads are misaligned to NUMT and the misalignment is biased toward one allele over another, this could affect the detection of heteroplasmy and estimation of heteroplasmy level.

We will address the chromosome 1 NUMT region, which is ~5000 bp long, in the next paragraph.  Except for this region, all other NUMT regions are much shorter than our typical insert size of 250-400bp and many are shorter than our read length of 100nt.  If a NUMT region is much shorter than 100bp, it is improbable for a 100nt read to misalign. Since the insert sizes of our data are much longer than the NUMT regions, except the chromosome 1 NUMT region, even if a read is misaligned to an incorrect genome, its mate (e.g., the other read in the paired-end sequencing product) is likely to be correctly mapped.  Since the aligner program BWA gives MAPQ=0 to a read if its mate is aligned to a different chromosome, such reads would be filtered out in our analysis.

For the chromosome 1 NUMT region, we performed the following analysis. We took all reads aligned to the chromosome 1 NUMT region and all reads aligned to the mitochondrial DNA, and realigned them to the mtDNA reference genome without the nuclear genome, and searched for heteroplasmy across the mtDNA genome.  The results were the same as in the original analyses.
